# In Silico Discovery of a Potential PI3Kα Inhibitor Incorporating an Imidazole Moiety for Breast Cancer Treatment

**DOI:** 10.3390/cimb48101055

**Published:** 2026-10-13

**Authors:** Wenqing Jia, Yukui Ma, Rong Huang, Xi He, Xianchao Cheng

**Affiliations:** 1 School of Chemistry and Chemical Engineering, Qilu Normal University, Jinan 250200, China; 2 School of Pharmacy, Binzhou Medical University, Yantai 264003, China; 3 Tianjin Key Laboratory on Technologies Enabling Development of Clinical Therapeutics and Diagnostics (Theranostics), School of Pharmacy, Tianjin Medical University, Tianjin 300070, China

**Keywords:** PI3Kα, PI3Kα inhibitor, breast cancer, scaffold hopping

## Abstract

Excessive activation of PI3Kα is prevalent in breast cancer, aberrantly activating the PI3K/Akt/mTOR pathway to drive tumor progression, thus rendering PI3Kα a vital therapeutic target. Two PI3Kα inhibitors (alpelisib and inavolisib) have been approved for clinical application. Inavolisib exerts a mutation-selective degradation mechanism and functions by directly eliminating aberrant proteins. To develop novel PI3Kα inhibitors, we employed multiple computational strategies including scaffold hopping based on the structure of inavolisib, -CDOCKER docking, Lipinski’s rule of five, ADMET prediction, molecular dynamics (MD) simulation and GLORYx metabolic prediction. These tools were used to assess candidate compounds for their predicted binding modes and binding affinity with PI3Kα, physicochemical profiles, pharmacokinetic properties, metabolic pathways and generated metabolites. Ultimately, M-288, a computationally prioritized candidate compound and imidazole derivative with favorable predicted properties, was identified. Its alanine moiety formed a H-bond with the key residue Gln859 in the binding pocket, and the oxygen atom on its carboxyl group established a salt bridge with non-conserved Arg770 to enhance binding affinity. M-288 also formed H-bonds with Asp933, Tyr836 and Val851, facilitating its stable binding to PI3Kα. This compound was predicted to occupy the ATP-binding pocket. Compared with other screened candidates, it exhibited low predicted toxicity and no inhibitory activity against major hepatic CYP450 isoforms, while satisfying drug-likeness criteria and possessing favorable hepatic metabolic clearance. Molecular dynamics simulations verified its stable binding profile. This study indicates that M-288 serves as a potential PI3Kα inhibitor and provides valuable references for subsequent discovery of PI3Kα inhibitors.

## 1. Introduction

Breast cancer ranks among the most prevalent malignant tumors affecting the female population worldwide [1]. Hormone receptor-positive and human epidermal growth factor receptor 2-negative (HR+/HER2−) breast cancers are the most common subtype, accounting for roughly 75% of all newly diagnosed breast cancer cases [2]. Clinical guidelines for patients with early-stage HR+/HER2- breast cancer (EBC) generally advocate surgical resection combined with adjuvant endocrine therapy (ET) [3,4]. Supplementary radiotherapy, chemotherapy or targeted therapies can be administered selectively in accordance with personalized risk stratification. The core of advanced breast cancer is to prolong the survival period and improve the quality of life, and the comprehensive strategy of systemic drug treatment is adopted. Combined with local treatment to alleviate symptoms, the targeted/endocrine/chemotherapy/immune scheme is also formulated according to molecular typing [5,6].

Breast cancer is stratified into four main molecular subgroups by HR and HER2 expression patterns, including HR+/HER2− (luminal A-type), HR+/HER2+ (luminal B-type), HR−/HER2+ (HER2-amplified), and HR−/HER2− (triple-negative). Approximately 40% of patients with the HR+/HER2− subtype harbor oncogenic *PIK3CA* variants [7]. This gene is responsible for producing the p110α catalytic subunit of PI3Kα, a key signaling protein controlling cellular proliferation, survival and metabolic activity [8].

The most prevalent *PIK3CA* hot-spot variants lie within the exon 20 catalytic domain (p.H1047R), while exon 9 helical-region mutations (p.E542K, p.E545K) rank second; these mutations continuously stimulate downstream signal transduction [9,10,11]. Clinical data reveal that metastatic HR+/HER2− breast cancer patients carrying *PIK3CA* mutations display shorter overall survival than wild-type patients (19.6 months vs. 23.5 months, *p* = 0.04) [12]. Clinical evidence supports the therapeutic value of blocking PI3Kα signaling, which has promoted the development of various selective PI3Kα inhibitor candidates.

**Figure 1:**
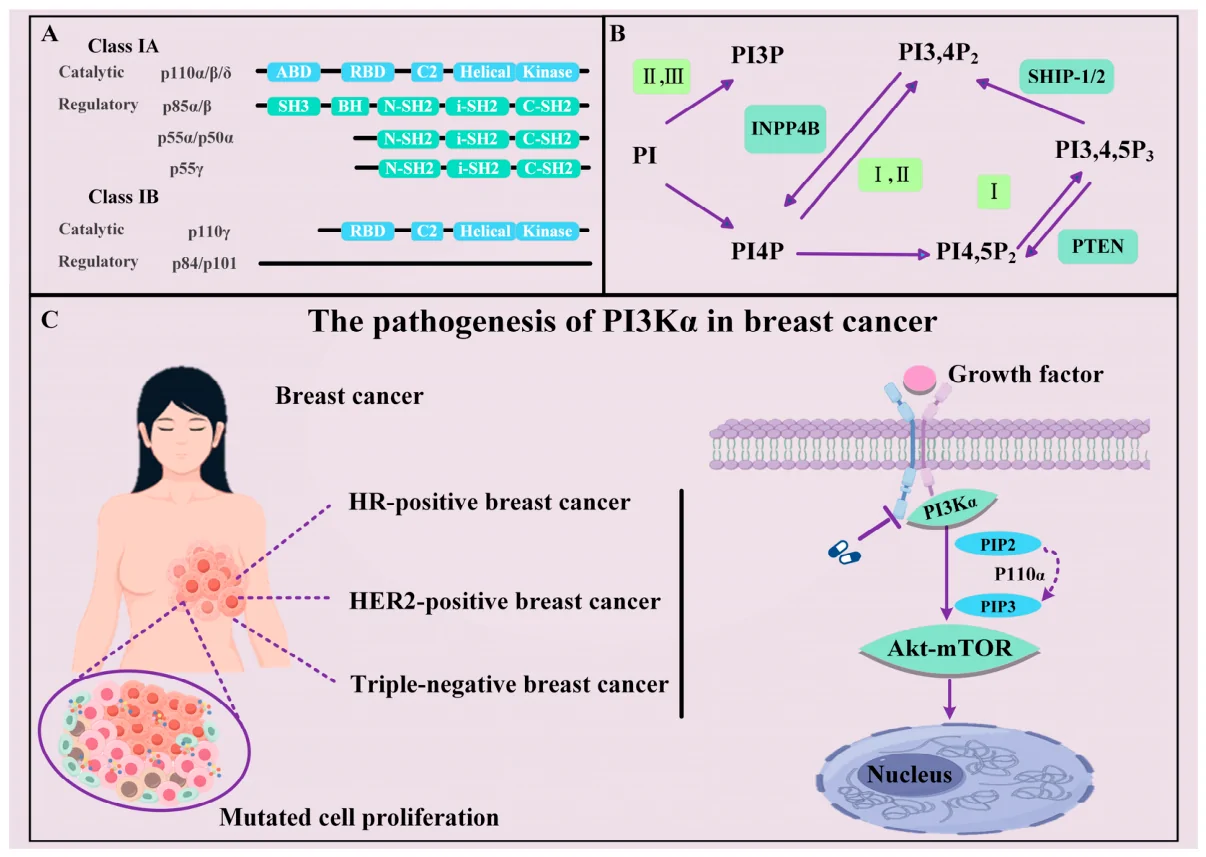
(**A**) The class I PI3K. (**B**) Schematic illustration depicting core synthetic and degradative routes responsible for generating phosphatidylinositol 3-phosphate (PI3P), phosphatidylinositol 3,4-bisphosphate (PI3,4P2), as well as phosphatidylinositol 3,4,5-trisphosphate (PI3,4,5P_3_, PIP3). (**C**) Diagram illustrating the pathogenic effects triggered by aberrant PI3Kα activity in breast cancer and the mechanism of action of selective PI3Kα inhibitors.

PI3K becomes activated downstream of growth factor receptors and integrins, and then modulates cellular proliferation, survival and motility via the PI3K/Akt/mTOR signaling cascade [13]. PI3K family proteins fall into three distinct classes. Class I PI3K consists of two polypeptides: a p85 regulatory subunit and a p110 catalytic subunit. The p110 has four isoforms (α, β, δ, γ; Figure 1A) [13,14]. The heterodimeric PI3Kα complex consists of p85α and p110α [15]. The *PIK3CA* gene coding for p110α frequently undergoes oncogenic hotspot alterations, which are detected in approximately 13% of all solid malignancies. These driver aberrations are especially prevalent in breast tumors: mutation frequencies reach 35–40% in HR+ cases and 25–30% for HER2+ subtypes [16,17,18].

The PI3K/Akt cascade serves as an essential signaling axis governing cellular survival and metabolic homeostasis [7,14]. Upon receiving upstream stimulatory inputs, PI3K catalyzes the phosphorylation of phosphatidylinositol 4,5-bisphosphate (PIP2) into phosphatidylinositol 3,4,5-trisphosphate (PIP3) (Figure 1B). Acting as an intracellular secondary messenger, PIP3 subsequently initiates activation of downstream signaling cascades. PIP3 interacts with the pleckstrin homology (PH) domain of Akt, recruiting Akt to the plasma membrane and inducing its conformational rearrangement; this process enhances the phosphorylation of Akt at Thr308 and Ser473, with PDK1 and mTORC2 acting as the core proteins driving these two phosphorylation events [19]. Once fully activated, Akt translocates from the cell membrane to the cytoplasm and nucleus, where it phosphorylates the mTORC1 negative regulators TSC2 and PRAS40 to activate mTORC1. Subsequently, mTORC1 phosphorylates its downstream targets p70S6K and 4EBP1, which are critical for cell proliferation and metabolism (Figure 1C).

PI3Kα inhibitors are highly selective targeted drugs against the abnormal overactivation of the PI3K/Akt/mTOR signaling pathway in breast cancer [20]. Alpelisib is the first approved PI3Kα inhibitor [21]. Its combination with fulvestrant serves as the standard second-line regimen for advanced HR+/HER2- breast cancer with *PIK3CA* mutations, significantly improving progression-free survival [22]. In addition, taselisib and inavolisib have further optimized inhibitory activity, capable of overcoming certain secondary drug-resistant mutations to alpelisib [23,24]. Some are also being explored in first-line therapy in combination with CDK4/6 inhibitors and endocrine drugs [23,24]. By specifically inhibiting the PI3Kα, these agents block the conversion of PIP2 to PIP3 and suppress Akt phosphorylation and downstream mTORC1 activation, thereby inhibiting tumor cell proliferation and reversing endocrine resistance [25,26]. The common adverse reactions include hyperglycemia, rash and diarrhea [27]. *PIK3CA* gene detection is required before administration, and close blood glucose monitoring is necessary during treatment, with generally good tolerability overall [27].

Among the known PI3Kα inhibitors, inavolisib displays potent PI3Kα subtype selectivity and can drive proteolysis to eliminate aberrant p110α protein variants. The selectivity for PI3Kα is more than 300 times that of other class I subtypes (β/δ/γ), accurately blocking the PI3K/Akt/mTOR pathway and reducing off-target toxicity [28,29]. It received FDA approval in October 2024 based on the INAVO120 trial, which evaluated a triplet combination of inavolisib (selective PI3Kα inhibitor with a dual mechanism of kinase inhibition and mutant p110α degradation) plus CDK4/6 inhibitor palbociclib plus the estrogen receptor antagonist fulvestrant for adults with *PIK3CA*-mutated, HR+/HER2- locally advanced or metastatic breast cancer after recurrence on or after adjuvant endocrine therapy. In the INAVO120 trial, this triplet regimen achieved a median progression-free survival (PFS) of 15.0 months at primary analysis versus 7.3 months with placebo plus palbociclib plus fulvestrant (HR 0.43; *p* < 0.0001), and updated data at 34.2 months of median follow-up showed a sustained benefit with a median PFS of 17.2 months [24,29]. Due to high selectivity, there is little interference with the β/δ/γ subtypes, and the incidence of adverse reactions such as hyperglycemia, rash, and diarrhea is significantly reduced [29]. In addition, researchers have initiated multiple clinical trials for inavolisib, including breast cancer (NCT07368998), *PIK3CA*-mutant cancers (NCT06496568), locally advanced and metastatic breast cancer (NCT07347600), and breast cancer (NCT05306041). Further trials containing inavolisib-containing regimens are currently being conducted for patients with the above indications [30,31,32].

The core goal of drug discovery is to generate novel molecules that act on disease-related targets for therapeutic benefits, with acceptable safety and drug-like physicochemical properties. This process includes lead discovery for potent molecules and lead optimization to improve efficacy and safety for clinical candidate screening. Scaffold-hopping can produce structurally new bioactive molecules while maintaining key properties such as target selectivity, activity and drug-likeness. However, novel druggable scaffolds are difficult to obtain, emphasizing the value of advanced scaffold-hopping strategies. Numerous scaffold hopping-derived compounds originate from marketed drugs, clinical candidates, bioactive small molecules and natural products. Some have received FDA approval, and others are in clinical trials. In silico design accounts for roughly 20% of modern drug discovery workflows [33].

To develop PI3Kα inhibitors with novel structures for breast cancer treatment, we optimized the structure of inavolisib via the scaffold hopping strategy, based on the interaction mechanism between inavolisib and the active pocket of PI3Kα. In this study, we successfully identified a computationally prioritized candidate PI3Kα inhibitor through a combination of scaffold hopping, molecular docking, pharmacokinetic evaluations using Swiss ADME and ADMETlab 3.0, toxicity predictions via Discovery Studio 3.5 and ProTox-3.0, metabolite analysis, and molecular dynamics simulations.

## 2. Materials and Methods

### 2.1. Scaffold Hopping

By leveraging structural innovation, scaffold hopping strikes an optimal balance between preserving biological activity and attaining molecular novelty [34]. As a critical tactic in drug discovery, it offers an effective solution to the “me-too” conundrum and propels the advancement of first-in-class drug candidates. Integrating this strategy with in silico calculation tools can markedly raise the success probability throughout drug discovery workflows. Hanan and colleagues have clarified how inavolisib occupies the ATP pocket of PI3Kα [28]. Aiming to create new PI3Kα inhibitors featuring superior therapeutic potency and safety profiles, the present study carried out structural modification of inavolisib via scaffold hopping on the basis of the intermolecular interaction pattern between this drug and the ATP-binding pocket of PI3Kα. The structural remodeling of inavolisib was performed in Discovery Studio 3.5 (Accelrys, San Diego, CA, USA) via scaffold hopping: firstly, appropriate sites for structural derivatization were identified; secondly, the built-in fragment library of the software was screened to generate candidate derivative molecules.

### 2.2. Virtual Screening

Virtual screening represents a routinely adopted strategy within drug discovery research. In this research, molecular docking-based virtual screening analyses were executed in Discovery Studio 3.5 (Accelrys, San Diego, CA, USA) [35]. Given the known active site, the “Define and Edit Binding Sites” function within Discovery Studio 3.5 was applied to precisely define the binding region of PI3Kα (PDB entry: 8EXV) [28]. Subsequently, the “From Current Selection” option was adopted to generate a spherical binding pocket near major functional residues (Arg770, Met772, Ser774, Pro778, Trp780, Ile800, Lys802, Tyr836, Ile848, Glu849, Val850, Val851, ARG852, ASN853, Ser854, His855, Thr856, Gln859, Met922, Phe930, Ile932, Asp933) within the ATP-binding site. The geometric parameters were optimized to a radius of 7.4 Å, with the center coordinates set at X = −17.16, Y = 13.14, Z = 29.16. Redocking results showed that inavolisib displayed high conformational consistency between the redocked pose and its crystal conformation, with an RMSD (0.4846 Å) of less than 1.0 Å (Figure S1). The low RMSD value (<1.0 Å) indicates that the CDOCKER protocol is reliable for subsequent molecular docking studies. In addition, the geometric parameters of PI3Kβ (PDB entry: 4BFR) were optimized to a radius of 6.76 Å, with the center coordinates set at X = 1.45, Y = 76.32, Z = 80.24. The geometric parameters of PI3Kδ (PDB entry: 2WXL) were optimized to a radius of 7.39 Å, with the center coordinates set at X = −9.31, Y = −30.75, Z = 23.75. The geometric parameters of PI3Kγ (PDB entry: 5G2N) were optimized to a radius of 11.33 Å, with the center coordinates set at X = 47.26, Y =13.63, Z = 32.23.

All candidate small-molecule compounds were preprocessed via the Prepare Ligands tool embedded in the Small Molecules module of Discovery Studio 3.5 (Accelrys, San Diego, CA, USA). Ligand conformational preparation covered multiple steps: ion state generation at pH 7.0 ± 2.0, desalting treatment, preservation of inherent stereochemistry from three-dimensional structures, and geometric optimization under the CHARMm force field framework. Subsequent molecular screening for all prepared compounds was accomplished using the CDOCKER algorithm. -CDOCKER energy is the total docking energy calculated, and its core function is to perform relative ranking for different ligands or conformations during the docking workflow rather than serving as a rigorous thermodynamic physical quantity.

### 2.3. Assessment of Drug-like Properties

This segment analyzed molecular structural features and pharmacokinetic behaviors via three computational tools: Discovery Studio 3.5 (Accelrys, San Diego, CA, USA), ADMETlab 3.0 (https://admetlab3.scbdd.com/, accessed on 20 March 2026) and SwissADME (https://www.swissadme.ch/, accessed on 21 March 2026). The assessment covered multiple critical physicochemical and pharmacokinetic indicators: molecular weight (MW), lipophilic coefficient (LogP), the number of rotatable bonds (nRot), H-bond acceptors (nHA), H-bond donors (nHD), topological polar surface area (TPSA), plasma clearance (CLplasma), half-life (T_1/2_), bioavailability score, skin permeability (Log *K*p), and volume distribution (VDss). Meanwhile, the toxicological profiles of candidate molecules were predicted through ProTox-3.0 (https://tox.charite.de/protox3/, accessed on 17 August 2026) and TOPKAT platforms (Accelrys, San Diego, CA, USA), covering indicators such as median lethal dose (LD_50_), female and male mouse NTP toxicity, Ames mutagenicity test results, DTP toxicity data, as well as skin irritation risk.

### 2.4. Molecular Dynamics Simulations

Molecular dynamics (MD) simulations were performed using GROMACS 2020.3 software. The amber99sb-ildn force field and the general Amber force field (GAFF) were used to generate the parameters and topologies of proteins and ligands, respectively. A simulation box was constructed with a minimum distance of over 1 nm between any protein atom and the box boundary, which was solvated with SPC216 water molecules and neutralized by adding Na^+^ and Cl^−^ ions. The entire system was optimized by the steepest descent method, so that the unreasonable contact or atom overlap in the system is reduced. To achieve sufficient pre-equilibration of the simulation system, the NVT ensemble and the NPT ensemble were performed for 100 ps at 300 K and 1 bar, respectively. Subsequently, the MD simulation of 100 ns was performed with periodic boundary conditions, and the temperature (300 K) and pressure (1 bar) were controlled by the V-rescale and Parrinello–Rahman methods, respectively. The leapfrog algorithm with a 2 fs timestep was employed, PME was used for long-range electrostatic calculations, and LINCS constrained all covalent bonds. RMSF was calculated from the trajectory with a sampling interval of 10 ps. The binding free energy of the compound was calculated by gmx_mmpbsa [36].

### 2.5. Prediction of Metabolic Regulatory Pathways and Related Metabolites

In this study, human metabolic prediction of the target compound was conducted using the GLORYx tool (https://nerdd.univie.ac.at/gloryx, accessed on 28 June 2026), which enables comprehensive prediction of both phase I and phase II metabolites. GLORYx generates putative metabolite structures according to systematic metabolic reaction rule sets and employs metabolism sites (SoMs) predicted by FAME 3 to score and rank all candidate metabolites. The predictions made by GLORYx are scored and ranked (per input molecule) based on the predicted SoM probabilities of the atoms involved in the reaction and a simple binary weighting of the corresponding reaction type. All predicted metabolites were ranked by priority score; a higher priority score indicates a higher relative probability of metabolite formation rather than experimental quantitative yield. Following file preparation, the SDF format file of M-288 was imported and submitted on the platform. Relevant output data were subsequently sorted out and systematically analyzed.

## 3. Results

### 3.1. Scaffold Hopping

In inavolisib, the carbonyl and amino groups on propanamide can form H-bonds with Gln859 and Ser854. Gln859 and Ser854 are key amino acids in the ATP-binding pocket, and PI3Kα inhibitors typically form H-bonds between these two conserved amino acids. The H-bonds between the fluorine atom on the difluoromethyl group and the non-conserved amino acid Ser774 enhances the targeting ability of the drug. The oxygen atom on oxazepine forms H-bonds with the key amino acid Val851 in the target. Val851 is also crucial for PI3Kα inhibitors to target p110α. In addition, the carbonyl group on oxazolidin-2-one can form a H-bond with Tyr836, further enhancing the targeting effect.

**Figure 2:**
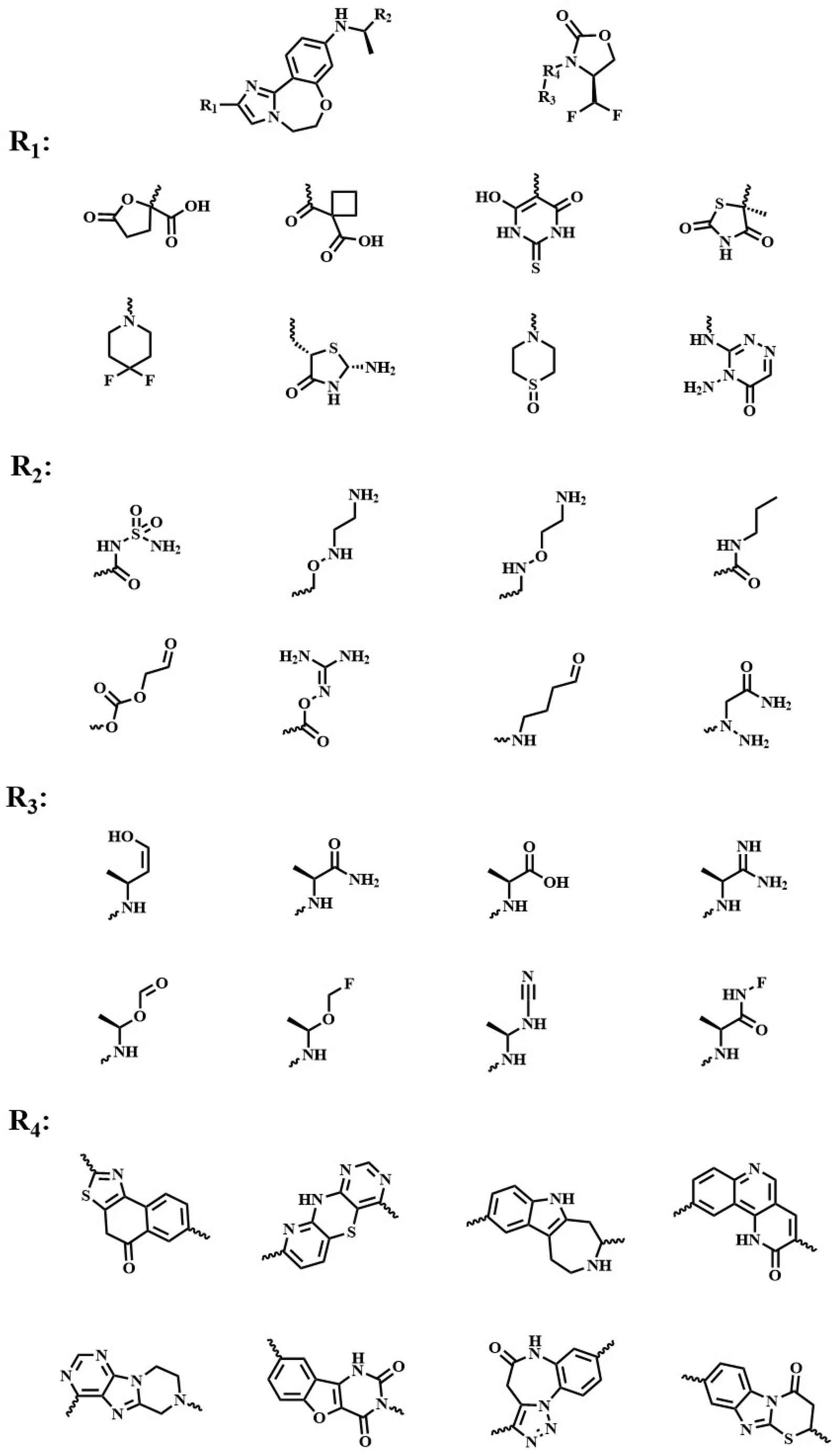
Partial results of scaffold hopping.

To develop novel PI3Kα inhibitors with higher targeting ability, we implemented two structural modification methods: (1) retaining the 5,6-dihydrobenzo[*f*]imidazo[1,2-*d*][1,4]oxazepine scaffold and performing scaffold hopping on R_1_ and R_2_; (2) retaining the (*S*)-4-(difluoromethyl)oxazolidin-2-one moiety and performing scaffold hopping on R_3_ and R_4_. H-bond interactions are essential for governing molecular binding affinity and maintaining stable conformational states of complexes. Therefore, when designing PI3Kα inhibitors capable of stably binding to the ATP active pocket, emphasis should be placed on the hydrogen bonding states formed with this pocket, while maintaining favorable safety and metabolic stability characteristics. Through structural optimization of inavolisib, we generated 299 novel compounds for further study (Figure 2).

### 3.2. Virtual Screening

Molecular docking simulations via the CDOCKER algorithm were further adopted to explore the interaction patterns between candidate compounds and the PI3Kα, from which 13 derivatives with superior binding capacity were screened out. The chemical structures and corresponding -CDOCKER energy values of these selected candidates are summarized in Table 1. Virtual screening results demonstrated that all 13 compounds possessed higher -CDOCKER energy. Of note, M-21, M-31, M-288, M-311, M-315, M-2170, M-329, M-338, M-352, M-360, M-2233, M-3105, and M-41 displayed -CDOCKER energies of 52.1583, 47.3240, 45.6221, 43.1438, 42.6272, 41.9005, 41.3046, 41.0815, 40.7549, 40.5123, 40.2314, 39.8378, and 39.0002 kcal/mol, respectively, surpassing those of inavolisib (37.6246 kcal/mol) and alpelisib (31.6270 kcal/mol).

**Figure 3:**
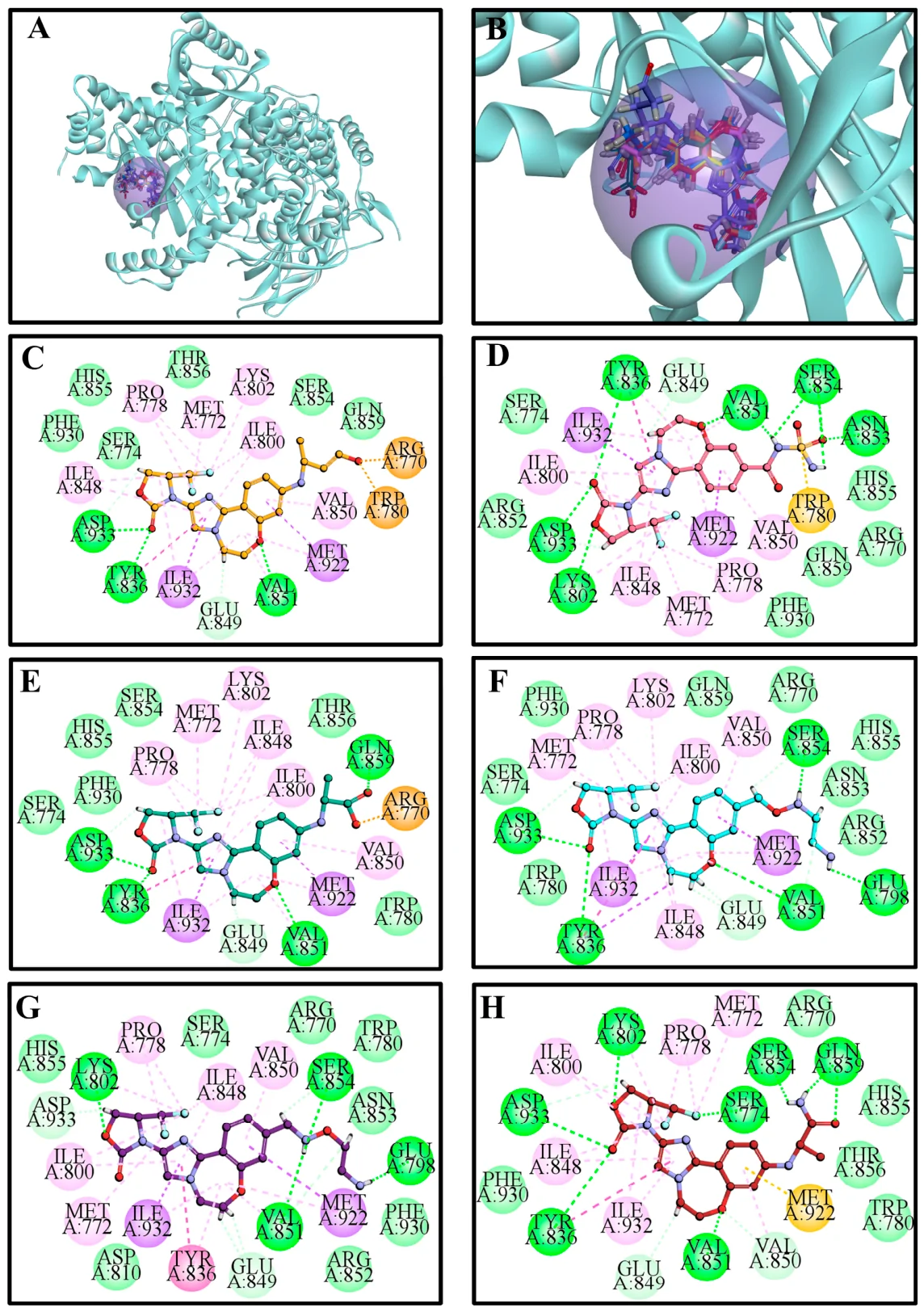
Analysis of the binding modes between compounds and PI3Kα. (**A**,**B**) The binding pocket (purple spherical shape) of ATP-competitive PI3Kα inhibitors. (**C**) The binding mode between M-21 and PI3Kα. (**D**) The binding mode between M-31 and PI3Kα. (**E**) The binding mode between M-288 and PI3Kα. (**F**) The binding mode between M-311 and PI3Kα. (**G**) The binding mode between M-315 and PI3Kα. (**H**) The binding mode between inavolisib and PI3Kα. Dark green dashed line: conventional hydrogen bond; light green dashed line: carbon hydrogen bond; pink/pale purple/purple/orange dashed line: pi interactions; dark orange dashed line: salt bridge.

Figure 3 illustrates the binding modes of M-21, M-31, M-288, M-311, M-315, and inavolisib at ATP-binding sites. Figure 3A,B show the PI3Kα and the binding pocket of PI3Kα inhibitors. As shown, all compounds formed H-bond interactions with Asp933, Glu849 and Val851 (Figure 3C–H). Additionally, M-21 formed H-bond interactions with Tyr836 (Figure 3C); M-31 formed H-bond interactions with Tyr836, Lys802, Ser854, and Asn853 (Figure 3D); M-288 formed H-bonds with Tyr836 and Gln859 (Figure 3E); M-311 formed H-bonds with Tyr836, Glu798 and Ser854 (Figure 3F); M-315 formed H-bonds with Lys802, Ser854, and Glu798 (Figure 3G); inavolisib formed H-bonds with Lys802, Ser854, Gln859, Tyr836 and Ser774 (Figure 3H). The superposition (Figure S2) of M-288 and inavolisib in the PI3Kα binding pocket showed that the two compounds occupied a highly overlapping binding space.

In addition, we also investigated the interactions between M-288 and three isoforms, PI3Kβ, PI3Kδ and PI3Kγ (Figure S3). The corresponding -CDOCKER energy values of M-288 and inavolisib binding to the ATP-binding pocket of PI3Kα/β/δ/γ are summarized in Table S1. M-288 can form H-bonds with LYS799 and LYS711 in PI3Kβ but exhibits no interactions with the key amino acids VAL848 and TRP781 of this target. The -CDOCKER energy values of M-288 and inavolisib for PI3Kβ are 33.1794 and 29.6114 kcal/mol, respectively, both of which are lower than their corresponding -CDOCKER energy values for PI3Kα. For the PI3Kδ, M-288 forms H-bonds with LYS779, with no interaction observed with the key amino acid VAL828. The -CDOCKER energy of M-288 (27.0735 kcal/mol) is comparable to that of inavolisib (27.7060 kcal/mol), and both values are lower than the -CDOCKER energy values obtained for PI3Kα. In the PI3Kγ, M-288 forms H-bonds with the key amino acid VAL882, while no interactions occur with other key residues including LYS833, ASP836, ASP841 and ASP964. The -CDOCKER energy values of M-288 and inavolisib for PI3Kγ are 36.5349 and 27.3720 kcal/mol, respectively; similarly, both values are lower than their respective -CDOCKER energy values for PI3Kα. These results demonstrate that M-288 establishes more extensive interactions with key amino acids of PI3Kα and shows preferable binding affinity toward PI3Kα over the other three isoforms.

### 3.3. Analysis of Drug-Likeness

**Figure 4:**
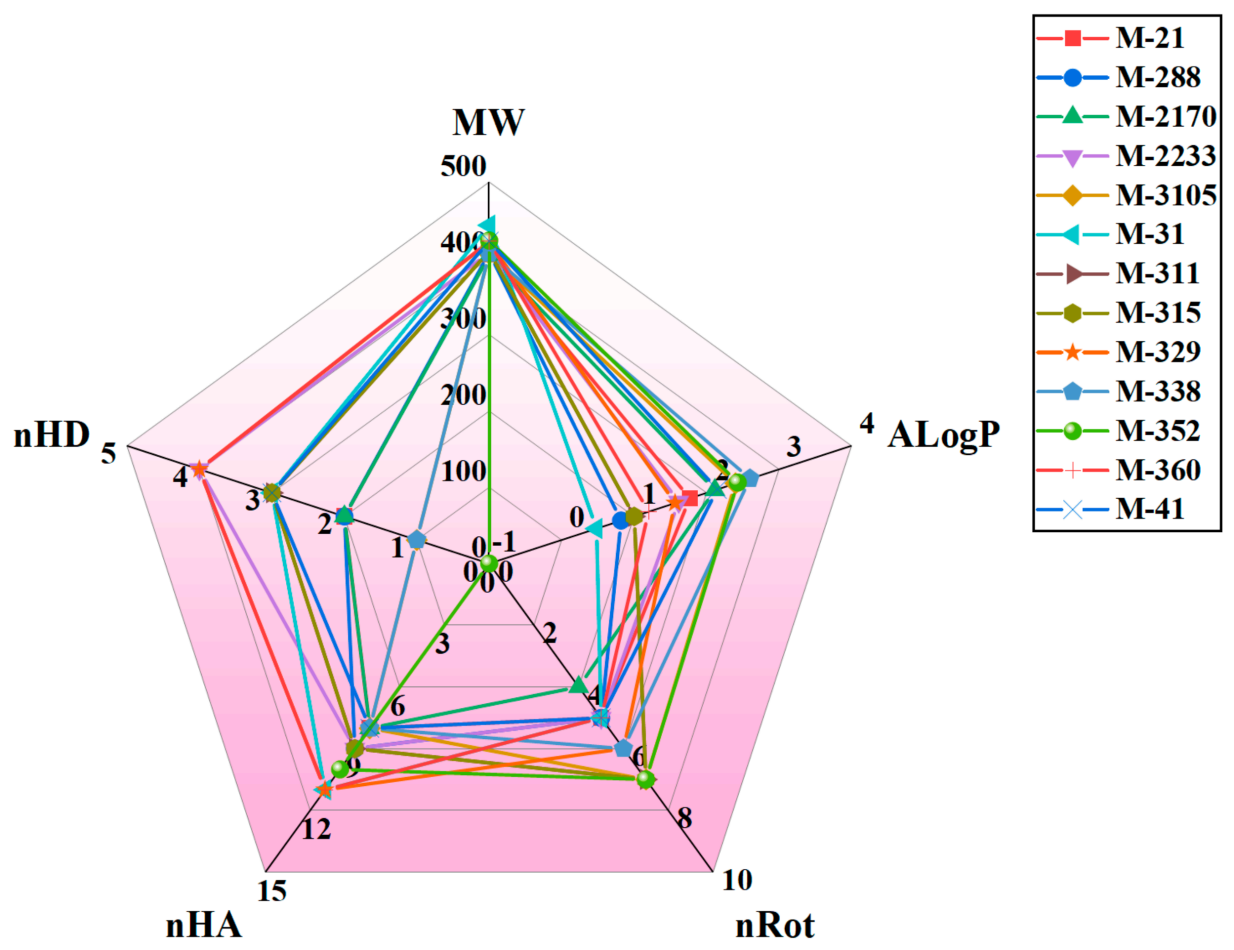
Radar plot-based evaluation of Lipinski’s Rule of Five.

**Figure 5:**
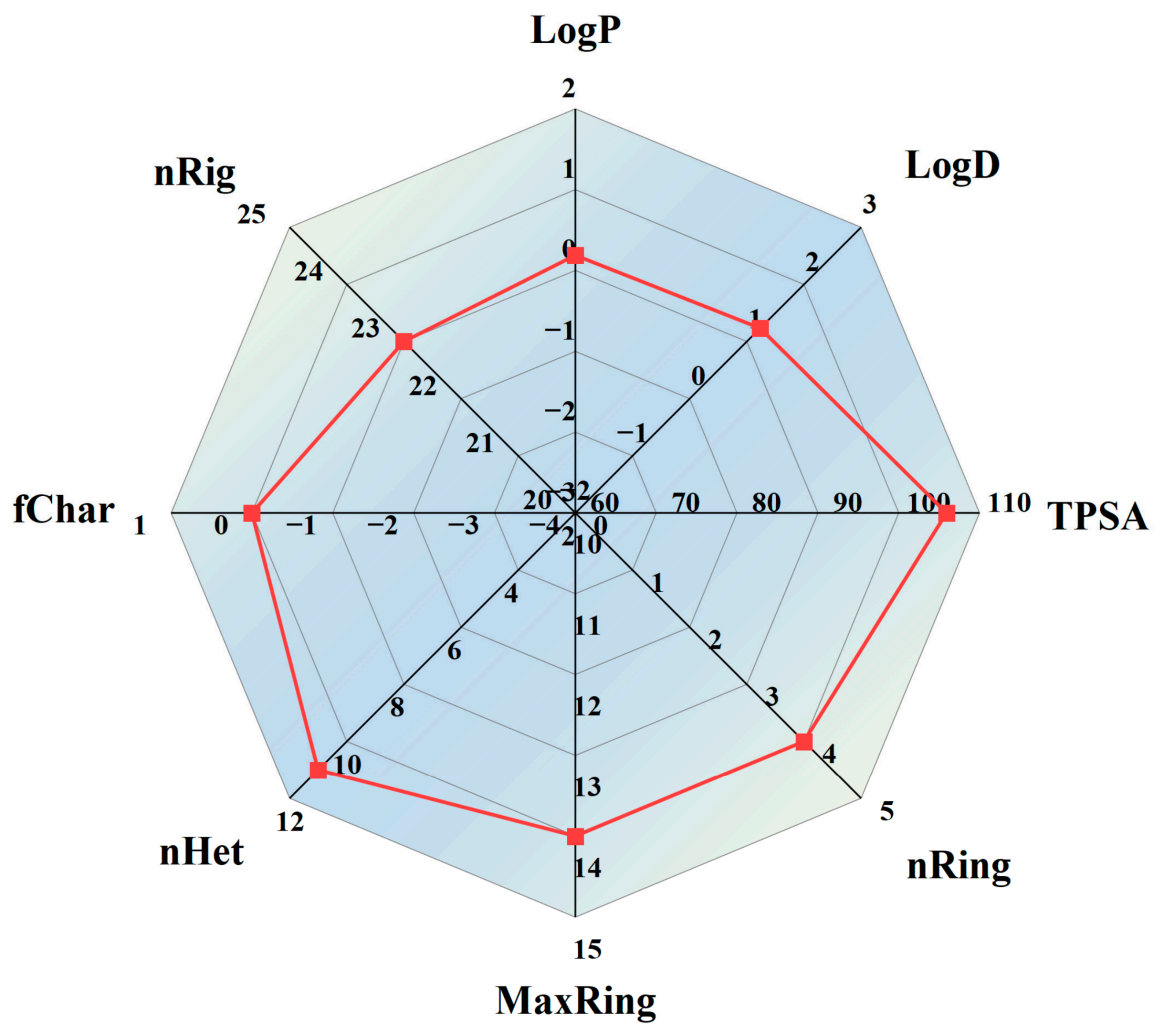
Analysis of physicochemical properties of M-288.

As illustrated in Figure 4 and Table S2, M-21, M-288, M-311, M-315, M-2170, M-338, M-352, M-2233, M-3105, and M-41 exhibited superior properties compared with M-31, M-329, and M-360 in terms of MW, ALogP, nRot, nHA, and nHD. ADMET-related properties including human intestinal absorption, water solubility, plasma protein binding (PPB), rodent carcinogenic risk, Ames mutagenicity, developmental toxicity potential (DTP prediction) and skin irritation risk were analyzed for the thirteen prioritized candidate molecules. All relevant data are summarized in Table 2 and Table 3. Oral bioavailability is predominantly governed by two critical indicators: human intestinal absorption capacity and aqueous solubility. The solubility classification values of these derivatives were grade 2 or grade 3, predicting that the majority of candidates meet the solubility criteria. The predicted intestinal absorption levels of the 13 compounds ranged from 0, 1 to 2. Aside from M-31 and M-360, most analogs were predicted to exhibit moderate or favorable intestinal absorption. Moreover, the predicting results of toxicity (mouse male NTP, mouse female NTP, ames prediction, DTP prediction, and skin irritancy) of the 13 molecules showed that M-21, M-288, M-2170, M-360, M-2233, M-3105 and inavolisib had no predicted risk of carcinogen, mutagenicity, DTP and skin irritancy (Table 3). Compounds M-21, M-288, M-2170, M-2233 and M-3105 were screened out by integrating Lipinski’s Five Rules with the ADMET results. We further evaluated their pharmacokinetic profiles using the ADMETlab 3.0 and SwissADME platforms. As shown in Table 4, the predicted clearance rates of all five compounds were below 5 mL/min/kg, classifying them as low-clearance compounds consistent with inavolisib, with M-288 exhibiting the lowest predicted clearance rate. Additionally, the predicted T_1/2_ of all the compounds ranged from 1 to 4 h, which categorized them as short half-life drugs. M-288 and inavolisib were predicted to have no inhibitory effect on cytochrome P450 (CYP450) enzymes including CYP2D6, CYP1A2, CYP2C19, CYP2C9 and CYP3A4. The predicted bioavailability score and Log *K*p of M-288 were 0.56 and −7.29 cm/s, respectively. The predicted VDss values of M-288 was 0.324 L/kg, indicating that M-288 was predicted to be primarily distributed in the blood with limited penetration into tissues. The predicted oral LD_50_ of M-288 was 1600 mg/kg, falling into the category of low-toxicity compounds. We further evaluated the drug-likeness of M-288 by ADMETlab 3.0, including LogP, LogD, TPSA, nRing, MaxRing, nHet, fChar and nRig (Figure 5). According to the obtained data, M-288 showed a favorable predicted profile.

### 3.4. Molecular Dynamics Simulation of M-288/Inavolisib-PI3Kα System

**Figure 6:**
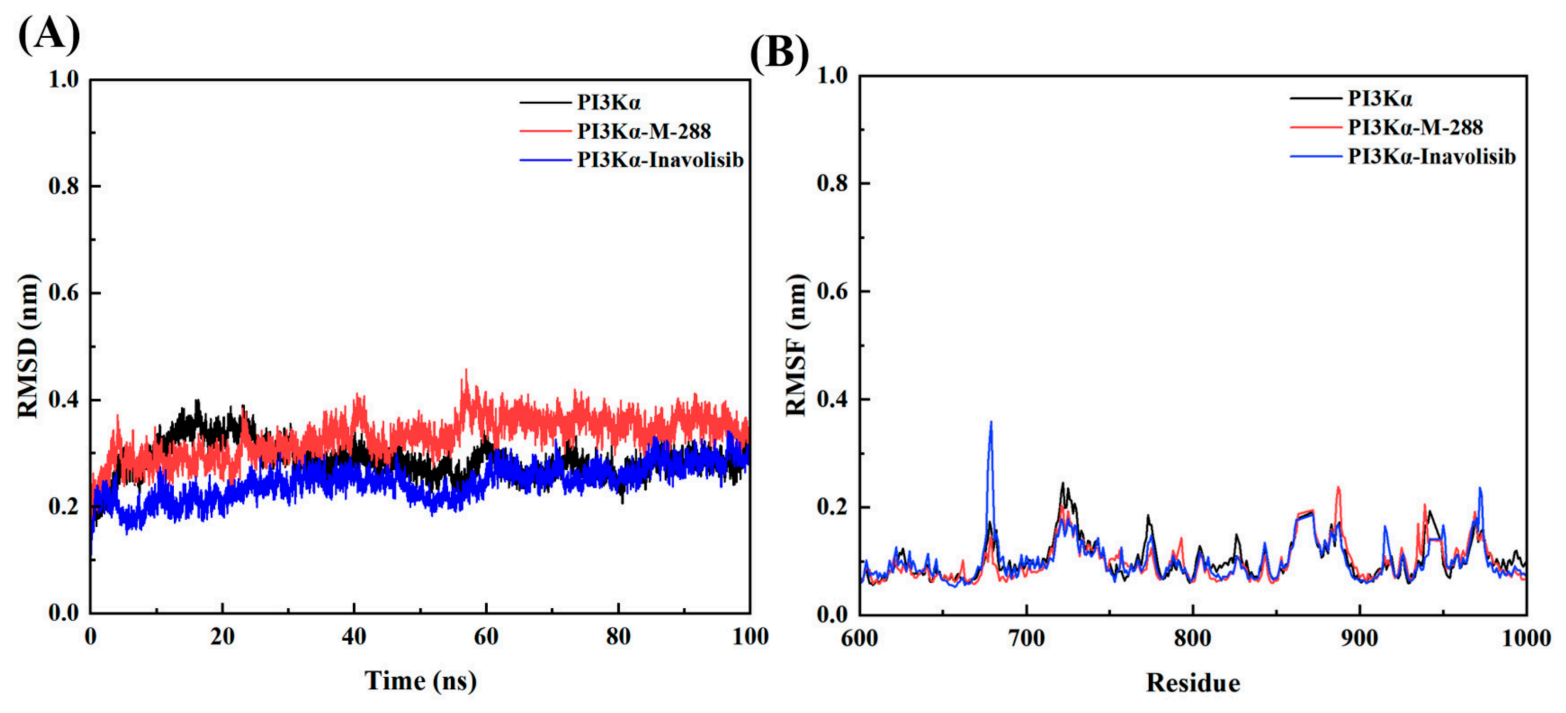
(**A**) The root mean square deviation (RMSD) of PI3Kα and PI3Kα-M-288/PI3Kα-inavolisib. (**B**) The root mean square fluctuation (RMSF) of PI3Kα and PI3Kα-M-288/PI3Kα-inavolisib.

Root mean square deviation (RMSD) serves as a critical evaluation indicator for the structural stability of molecular dynamic simulations. A stable RMSD trend represents the completion of system equilibrium and validates the stability of the simulation system [37]. As shown in Figure 6A, PI3Kα, PI3Kα-M-288 and PI3Kα-inavolisib reached equilibrium after 10 ns, with average RMSD values of 0.291 nm, 0.336 nm and 0.251 nm, respectively. These results indicate that simulation systems achieved stable conformational equilibrium.

Root mean square fluctuation (RMSF) is a core parameter in molecular dynamics (MD) simulations, which is adopted to measure the average fluctuation amplitude of each amino acid residue of proteins throughout the whole simulation period. Higher RMSF values correspond to greater flexibility (stronger fluctuation) at the corresponding residue site, whereas lower values indicate a more stable protein region [38,39,40]. As illustrated in Figure 6B, the average RMSF of the PI3Kα-M-288 complex reached 0.115 nm, while the PI3Kα-inavolisib protein exhibited a value of 0.119 nm.

**Figure 7:**
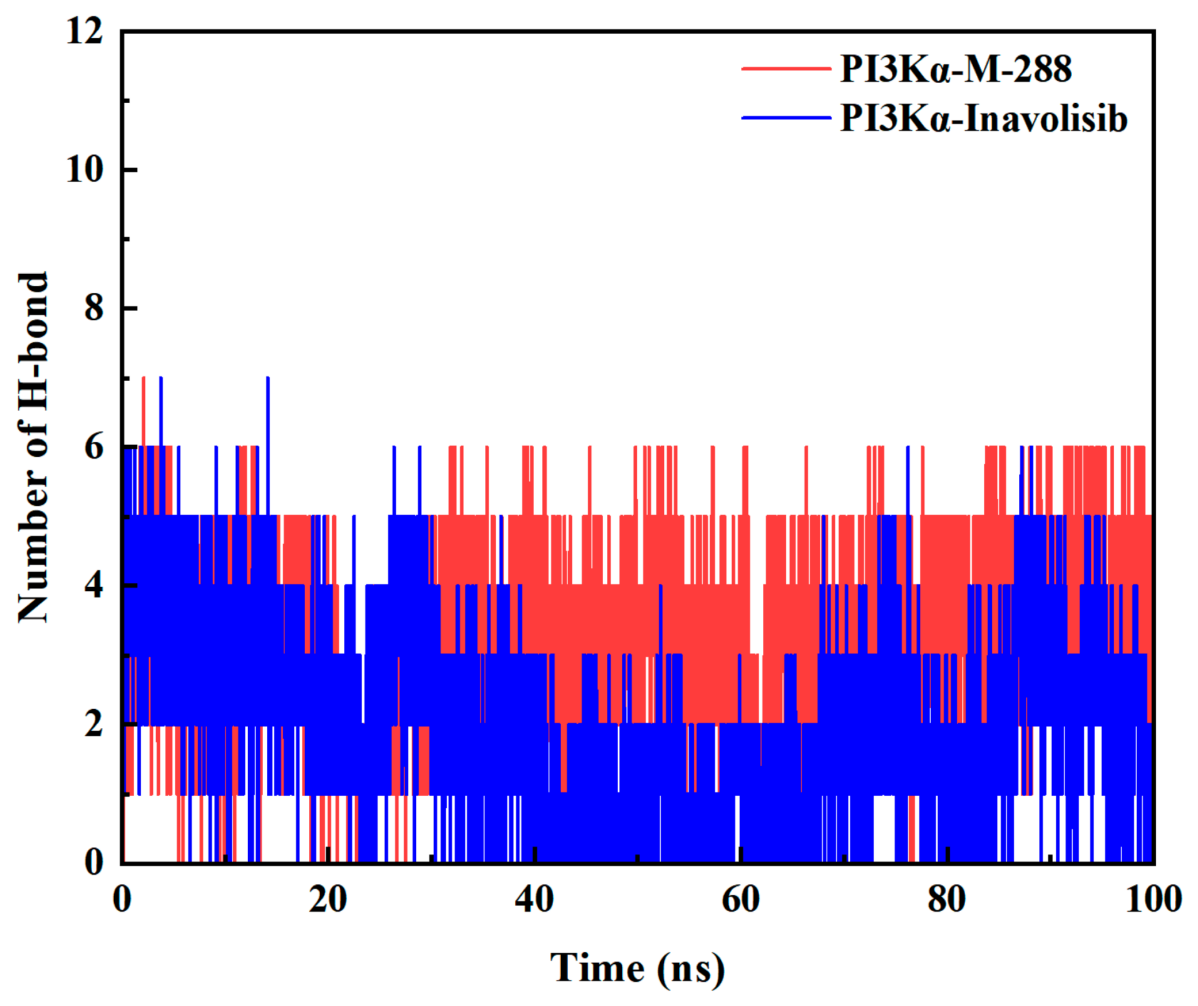
The curve of the number of H-bonds of PI3Kα-M-288/PI3Kα-inavolisib during 100 ns simulations.

To explore intermolecular contacts between the protein and ligand, the number of H-bonds were calculated based on the entire molecular dynamics trajectory after simulation equilibration. As shown in Figure 7, M-288 and inavolisib formed an average of 3.22 and 2.05 H-bonds with PI3Kα, respectively. This demonstrates persistent and stable H-bond contacts within the complexes. Such H-bond interactions are indispensable for M-288 to selectively recognize and bind to the PI3Kα.

To clarify intermolecular binding energies between target proteins and small-molecule ligands, the gmx_mmpbsa tool script was adopted to compute binding free energies of PI3Kα-M-288/inavolisib under equilibrated simulation states. Within MMPBSA decomposition calculations, overall binding free energy is split into four separate energetic contributions: electrostatic forces, van der Waals contacts, polar solvation energy, and nonpolar solvation energy. All binding energy data for M-288/inavolisib are compiled in Table S3. Total binding free energies of −93.473 kJ/mol and −87.720 kJ/mol were obtained for the PI3Kα–M-288 and PI3Kα–inavolisib complexes, respectively, indicating favorable binding interactions between PI3Kα and these two compounds.

**Figure 8:**
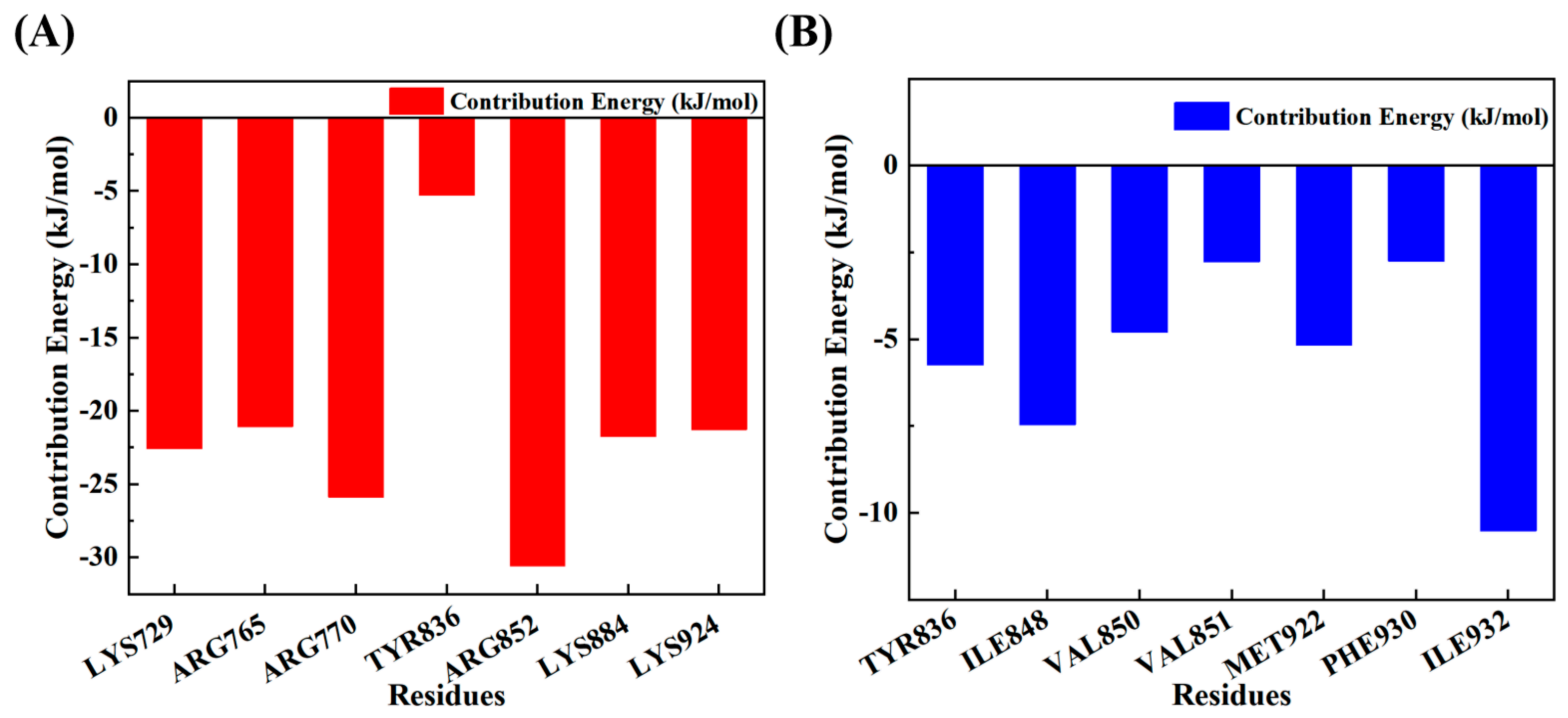
(**A**) Residual contribution energies of the interaction between PI3Kα and M-288. (**B**) Residual contribution energies of the interaction between PI3Kα and inavolisib.

To further explore the key amino acids responsible for protein–small molecule interactions, we performed energy decomposition analysis on the binding free energy. The results revealed that residues Lys729, Arg765, Arg770, Tyr836, Arg852, Lys884 and Lys924 were the major interacting residues for the PI3Kα–M-288 complex (Figure 8A), while Tyr836, Ile848, Val850, Val851, Met922, Phe930 and Ile932 constituted the key interacting residues for the PI3Kα–inavolisib complex (Figure 8B).

To investigate how different ligand-bound states affect protein conformational dynamics, dynamic cross-correlation maps (DCCMs) were computed for all systems (Figure S4). All systems displayed strong red positive correlations on the main diagonal, corresponding to residue autocorrelation and local cooperative movements. However, long-range off-diagonal correlation patterns differed greatly. The PI3Kα showed evenly distributed red and blue signals with weak long-range positive correlations, implying a loose structure lacking long-range residue cooperativity. In contrast, the M-288 and inavolisib systems displayed greatly strengthened cooperative motion networks, with cross-shaped deep-red positive correlation patches at residues 200–300, 400–500 and 600–700, indicating ligand binding induced strong inter-domain coupled motions and increased protein rigidity. Subtle differences existed between M-288 and inavolisib systems: while both retained high core-region correlations, the inavolisib system had stronger off-diagonal negative correlations (residues ~200 vs. ~600), suggesting greater reciprocal inter-domain motion and conformational tension. Collectively, DCCM results reveal that binding of M-288 and inavolisib remodeled the dynamic features of the protein, strengthening long-range residue cooperativity and stabilizing compact, functionally relevant conformations.

### 3.5. Prediction of Metabolic Pathways and Metabolites of M-288

**Figure 9:**
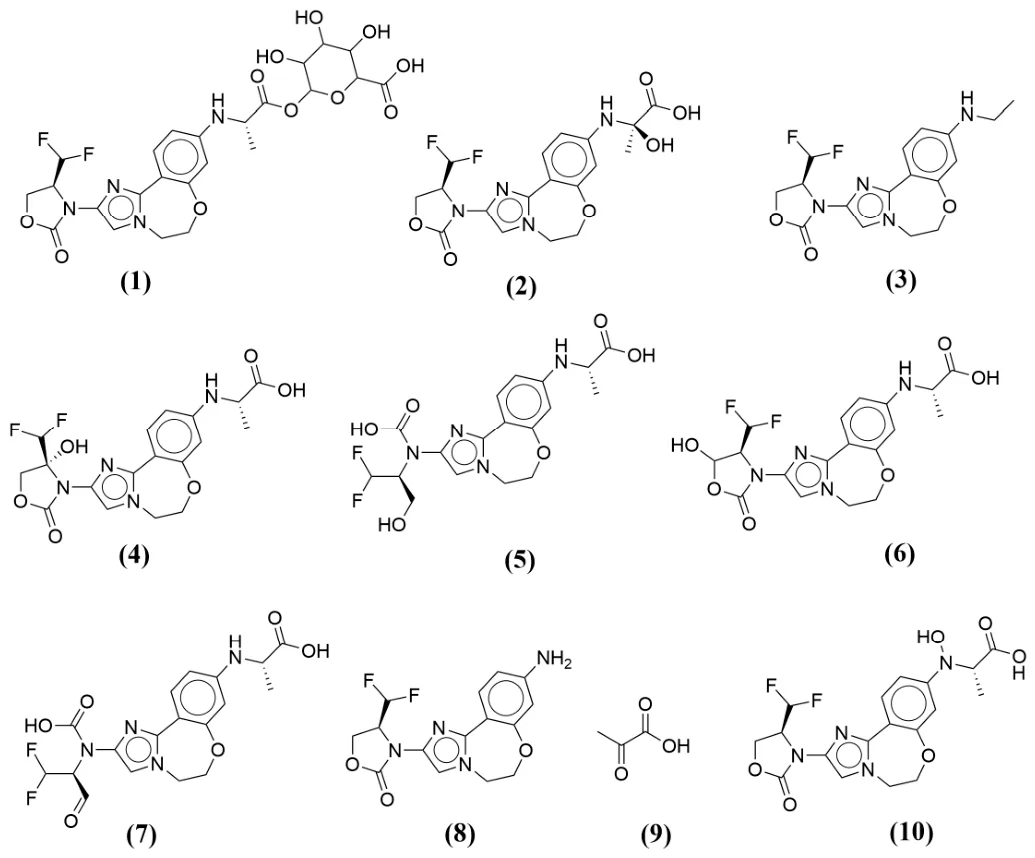
Metabolites (**1**–**10**) of M-288.

Metabolic prediction showed that M-288 might undergo Phase I/II metabolic transformations in the body, covering key pathways such as O-glucuronidation, aliphatic_hydroxylation, decarboxylation, hydrolysis, oxidative ether cleavage to one alcohol and one aldehyde/ketone, N-dealkylation, and so on (Figure 9). A total of 42 metabolites are generated through the above metabolic pathways, among which 10 metabolites have priority scores (Table S4) greater than 0.159, namely 6-(((2-((S)-4-(difluoromethyl)-2-oxooxazolidin-3-yl)-5,6-dihydrobenzo[f]imidazo[1,2-d][1,4]oxazepin-9-yl)-L-alanyl)oxy)-3,4,5-trihydroxytetrahydro-2H-pyran-2-carboxylic acid (**1**), (R)-2-((2-((S)-4-(difluoromethyl)-2-oxooxazolidin-3-yl)-5,6-dihydrobenzo[f]imidazo[1,2-d][1,4]oxazepin-9-yl)amino)-2-hydroxypropanoic acid (**2**), (S)-4-(difluoromethyl)-3-(9-(ethylamino)-5,6-dihydrobenzo[f]imidazo[1,2-d][1,4]oxazepin-2-yl)oxazolidin-2-one (**3**), (2-((R)-4-(difluoromethyl)-4-hydroxy-2-oxooxazolidin-3-yl)-5,6-dihydrobenzo[f]imidazo[1,2-d][1,4]oxazepin-9-yl)-L-alanine (**4**), (2-(carboxy((S)-1,1-difluoro-3-hydroxypropan-2-yl)amino)-5,6-dihydrobenzo[f]imidazo[1,2-d][1,4]oxazepin-9-yl)-L-alanine (**5**), (2-((4S)-4-(difluoromethyl)-5-hydroxy-2-oxooxazolidin-3-yl)-5,6-dihydrobenzo[f]imidazo[1,2-d][1,4]oxazepin-9-yl)-L-alanine (**6**), (2-(carboxy((S)-1,1-difluoro-3-oxopropan-2-yl)amino)-5,6-dihydrobenzo[f]imidazo[1,2-d][1,4]oxazepin-9-yl)-L-alanine (**7**), (S)-3-(9-amino-5,6-dihydrobenzo[f]imidazo[1,2-d][1,4]oxazepin-2-yl)-4-(difluoromethyl)oxazolidin-2-one (**8**), 2-oxopropanoic acid (**9**), and N-(2-((S)-4-(difluoromethyl)-2-oxooxazolidin-3-yl)-5,6-dihydrobenzo[f]imidazo[1,2-d][1,4]oxazepin-9-yl)-N-hydroxy-L-alanine (**10**). The priority scores are 0.984, 0.248, 0.248, 0.162, 0.162, 0.162, 0.162, 0.159, 0.159, and 0.159, respectively. Among them, metabolite 1 produced through O-glucuronidation was predicted to be the predominant metabolite.

## 4. Discussion

To develop more effective therapeutics for breast cancer, we implemented a scaffold hopping strategy to structurally modify inavolisib based on its interaction mechanism with PI3Kα. Using an integrated computational approach combining molecular docking, ADME evaluation (Discovery studio, ADMETLab 3.0 and SwissADME), toxicity prediction (ProTox-3.0 and Discovery studio), molecular dynamics simulations, and metabolite analysis (GLORYx), we systematically characterized: (1) predicted ligand–protein binding modes, (2) predicted compound pharmacokinetic properties, (3) predicted binding stability, and (4) predicted metabolic pathways and metabolites.

M-288 exhibits rational structural design and superior properties. Oxazolidin-2-one forms H-bonds with residues Asp933 and Tyr836. 1,4-oxazepane forms H-bonds with the key residue Val851. The carboxyl group on alanine can also form H-bonds with the key amino acid Gln859 in the binding site. Notably, the oxygen atom forms a salt bridge with the non-conserved amino acid Arg770, enhancing the binding affinity between the drug and the target. Such diverse intermolecular interactions are predicted to allow M-288 to bind tightly to PI3Kα. It is predicted to undergo hepatic metabolism for its efficient elimination. M-288 has a distinct structure from inavolisib. Similar to inavolisib, M-288 is predicted to form H-bonds with key amino acid residues Gln859, Val851 and Tyr836 in the binding pocket. During the 100 ns molecular dynamics simulation, M-288 and inavolisib formed an average of 3.22 and 2.05 H-bonds with PI3Kα, respectively. These results indicate persistent and stable H-bond contacts between M-288 and PI3Kα. The total binding free energies predicted for PI3Kα–M-288 (−93.473 kJ/mol) and PI3Kα–inavolisib (−87.720 kJ/mol) reflect favorable binding of both ligands to PI3Kα. M-288 was obtained by applying the scaffold-hopping strategy to inavolisib and selecting R_1_–R_4_ modification sites. This demonstrates that the structural modification strategy serves as a computational approach to rapidly identify predicted potential lead compounds.

Scaffold hopping combined with virtual screening serves as a pivotal methodology in contemporary rational drug design, particularly for the research and development of kinase-targeted inhibitors. Subsequent computational verification demonstrates that M-288 is predicted to anchor within the ATP-binding pocket of PI3Kα, which may confer this compound with promising putative inhibitory capacity against PI3Kα kinase function. Although this in silico technology provides support for drug discovery, the current study is limited to computational validation only. The biological activity, selectivity, pharmacokinetic properties, toxicity, and metabolic behavior of M-288 remain to be experimentally validated. In the future, we will further supplement ligand-based methodological analyses, including pharmacophore modeling and ligand similarity analysis based on the well-characterized PI3Kα inhibitor inavolisib, to further clarify the rationality of compound screening. Meanwhile, we will continue to explore the inhibitory activity of M-288 against PI3Kα and reveal its in vitro and in vivo anti-tumor mechanisms through CCK8 assay, Annexin V/PI double staining, Western blot assay, etc., in order to provide sufficient preclinical research data for its further development.

## 5. Conclusions

In this study, a scaffold hopping strategy was utilized to design novel PI3Kα inhibitors based on inavolisib. Through multi-level computational evaluations, including molecular docking, ADMET and toxicity prediction, molecular dynamics simulation, and metabolite analysis, M-288 was identified as a promising candidate. M-288 can stably bind to PI3Kα with sustainable H-bond interactions and favorable druggability. This study demonstrates the effectiveness of scaffold hopping in rapid lead compound discovery. Further systematic in vitro and in vivo experiments are required to validate its inhibitory activity and clinical potential.

## Figures and Tables

**Table 1:** Molecular structures and docking results.

ID Number	Chemical Structure	Molecular Formula	-CDOCKER Energy (kcal/mol)
M-21	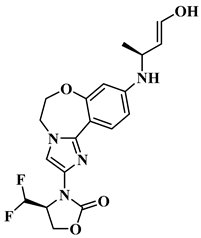	C_19_H_20_F_2_N_4_O_4_	52.1583
M-31	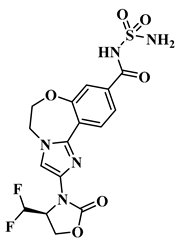	C_16_H_15_F_2_N_5_O_6_S	47.3240
M-288	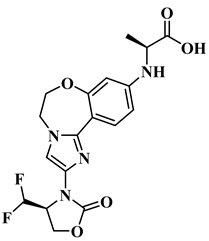	C_18_H_18_F_2_N_4_O_5_	45.6221
M-311	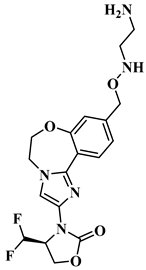	C_18_H_21_F_2_N_5_O_4_	43.1438
M-315	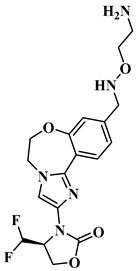	C_18_H_21_F_2_N_5_O_4_	42.6272
M-2170	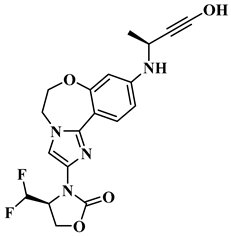	C_19_H_20_F_2_N_4_O_4_	41.9005
M-329	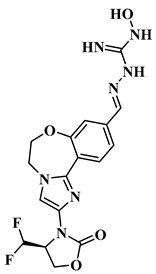	C_17_H_17_F_2_N_7_O_4_	41.3046
M-338	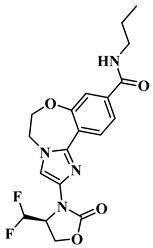	C_19_H_20_F_2_N_4_O_4_	41.0815
M-352	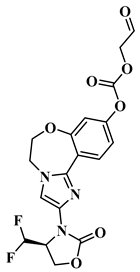	C_18_H_15_F_2_N_3_O_7_	40.7549
M-360	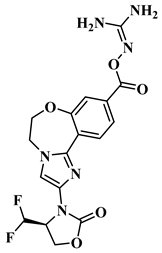	C_17_H_16_F_2_N_6_O_5_	40.5123
M-2233	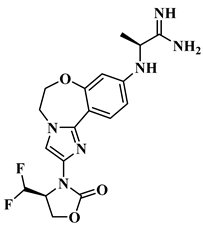	C_18_H_20_F_2_N_6_O_3_	40.2314
M-3105	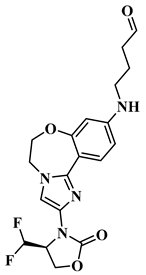	C_19_H_20_F_2_N_4_O_4_	39.8378
M-41	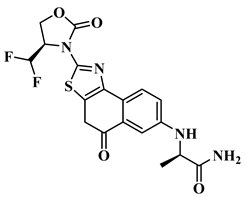	C_18_H_16_F_2_N_4_O_4_S	39.0002
Inavolisib	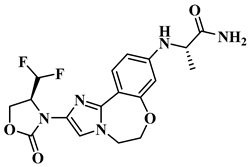	C_18_H_19_F_2_N_5_O_4_	37.6246
Alpelisib	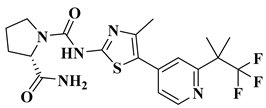	C_19_H_22_F_3_N_5_O_2_S	31.6270

**Table 2:** ADME property prediction analysis for candidates.

ID Number	Solubility Level ^1^	Absorption Level ^2^	PPB ^3^	PSA2D ^4^
M-21	3	0	True	85.233
M-31	2	2	True	146.375
M-288	3	0	True	102.534
M-311	2	0	True	103.403
M-315	2	0	True	103.403
M-2170	2	0	True	85.233
M-329	2	1	False	135.969
M-338	2	0	True	85.233
M-352	3	0	False	107.584
M-360	2	2	False	145.757
M-2233	2	0	False	123.274
M-3105	3	0	True	85.233
M-41	2	0	False	114.796
Inavolisib	2	0	True	111.773

^1^ Solubility Level: 1: very low, 2: yes, low, 3: yes, good, 4: optimal; ^2^ Absorption Level: 0: good, 1: moderate, 2 low, 3: very poor; ^3^ PPB: False: ≥90%, True: ≤90%; ^4^ PSA2D: ≤140.

**Table 3:** The TOPKAT predictions for candidates.

ID Number	Mouse Female NTP	Mouse Male NTP	Ames Prediction	DTP Prediction	Skin Irritancy
M-21	Non-Carcinogen	Non-Carcinogen	Non-Mutagen	Non-Toxic	None
M-31	Non-Carcinogen	Non-Carcinogen	Non-Mutagen	Toxic	None
M-288	Non-Carcinogen	Non-Carcinogen	Non-Mutagen	Non-Toxic	None
M-311	Non-Carcinogen	Non-Carcinogen	Non-Mutagen	Toxic	None
M-315	Non-Carcinogen	Carcinogen	Non-Mutagen	Toxic	None
M-2170	Non-Carcinogen	Non-Carcinogen	Non-Mutagen	Non-Toxic	None
M-329	Non-Carcinogen	Non-Carcinogen	Mutagen	Toxic	None
M-338	Non-Carcinogen	Non-Carcinogen	Non-Mutagen	Toxic	None
M-352	Carcinogen	Non-Carcinogen	Non-Mutagen	Non-Toxic	None
M-360	Non-Carcinogen	Non-Carcinogen	Non-Mutagen	Non-Toxic	None
M-2233	Non-Carcinogen	Non-Carcinogen	Non-Mutagen	Non-Toxic	None
M-3105	Non-Carcinogen	Non-Carcinogen	Non-Mutagen	Non-Toxic	None
M-41	Non-Carcinogen	Non-Carcinogen	Mutagen	Non-Toxic	None
Inavolisib	Non-Carcinogen	Non-Carcinogen	Non-Mutagen	Non-Toxic	None

DTP: developmental toxicity potential.

**Table 4:** The ADME properties predictions of M-21, M-288, M-2170, M-2233, M-3105 and inavolisib.

	M-21	M-288	M-2170	M-2233	M-3105	Inavolisib
PPB ^1^ (%)	82.8%	81.7%	85.5%	56.1%	83.4%	69.8%
CL_plasma_ ^2^ (mL/min/kg)	3.408	1.101	2.624	2.937	4.536	3.407
T_1/2_ ^3^ (h)	1.819	2.407	2.208	1.979	1.178	1.807
Bioavailability score ^4^	0.55	0.56	0.55	0.55	0.55	0.55
Log *K*p ^5^ (cm/s)	−6.63	−7.29	−6.50	−7.67	−7.42	−7.74
VDss ^6^ (L/kg)	0.956	0.324	0.849	1.352	0.756	1.136
CYP2D6 inhibitor ^7^	Yes	No	Yes	No	Yes	No
CYP1A2 inhibitor ^7^	No	No	No	No	No	No
CYP2C19 inhibitor ^7^	No	No	No	No	Yes	No
CYP2C9 inhibitor ^7^	Yes	No	Yes	No	Yes	No
CYP3A4 inhibitor ^7^	No	No	No	No	Yes	No

^1^ PPB: optimal: <90%. ^2^ CL_plasma_: >15 mL/min/kg: high clearance; 5–15 mL/min/kg: moderate clearance; <5 mL/min/kg: low clearance. ^3^ T_1/2_: ultra-short half-life drugs: T_1/2_ < 1 h; short half-life drugs: T_l/2_ between 1 and 4 h; intermediate short half-life drugs: T_l/2_ between 4 and 8 h; long half-life drugs: T_l/2_ > 8 h. ^4^ Bioavailability score: probability of F > 10% in rat. ^5^ Log *K*p: skin permeation value. ^6^ VDss: volume distribution; optimal: 0.04–20 L/kg. ^7^ CYP2D6 inhibitor, CYP1A2 inhibitor, CYP2C19 inhibitor, CYP2C9 inhibitor and CYP3A4 inhibitor: ‘No’ represents no inhibitory effect on the enzyme and ‘Yes’ indicates an inhibitory effect on the enzyme.

## Data Availability

The original contributions presented in this study are included in the article/Supplementary Materials. Further inquiries can be directed to the corresponding author.

## Supplementary Materials

The following supporting information can be downloaded at: https://www.mdpi.com/article/10.3390/cimb48101055/s1.
