# Supplementary material for: In Silico Discovery of a Potential PI3Kα Inhibitor Incorporating an Imidazole Moiety for Breast Cancer Treatment
Source: Curr Issues Mol Biol. 2026 Oct 13;48(10):1055. doi: 10.3390/cimb48101055 (PMC13703664; doi:10.3390/cimb48101055)
Supplement: Supplementary file 1 [file cimb-48-01055-s001.zip › cimb-4547567-supplementary.pdf]

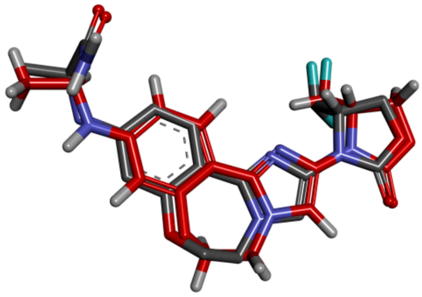

Figure S1. The docking pose of inavolisib overlaps with the crystallographic ligand pose. The red stick represents the docked structure of inavolisib, and the gray stick corresponds to the original inavolisib structure in the protein crystal.

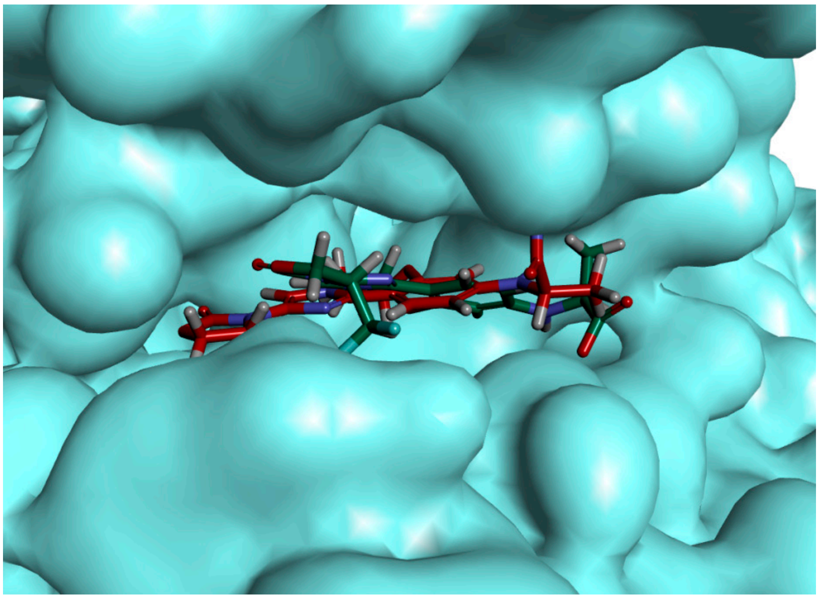

Figure S2. Superposition of M-288 and inavolisib in the binding pocket of PI3Kα. The protein is displayed as a cyan surface representation. M-288 is shown in green, and inavolisib is shown in red.

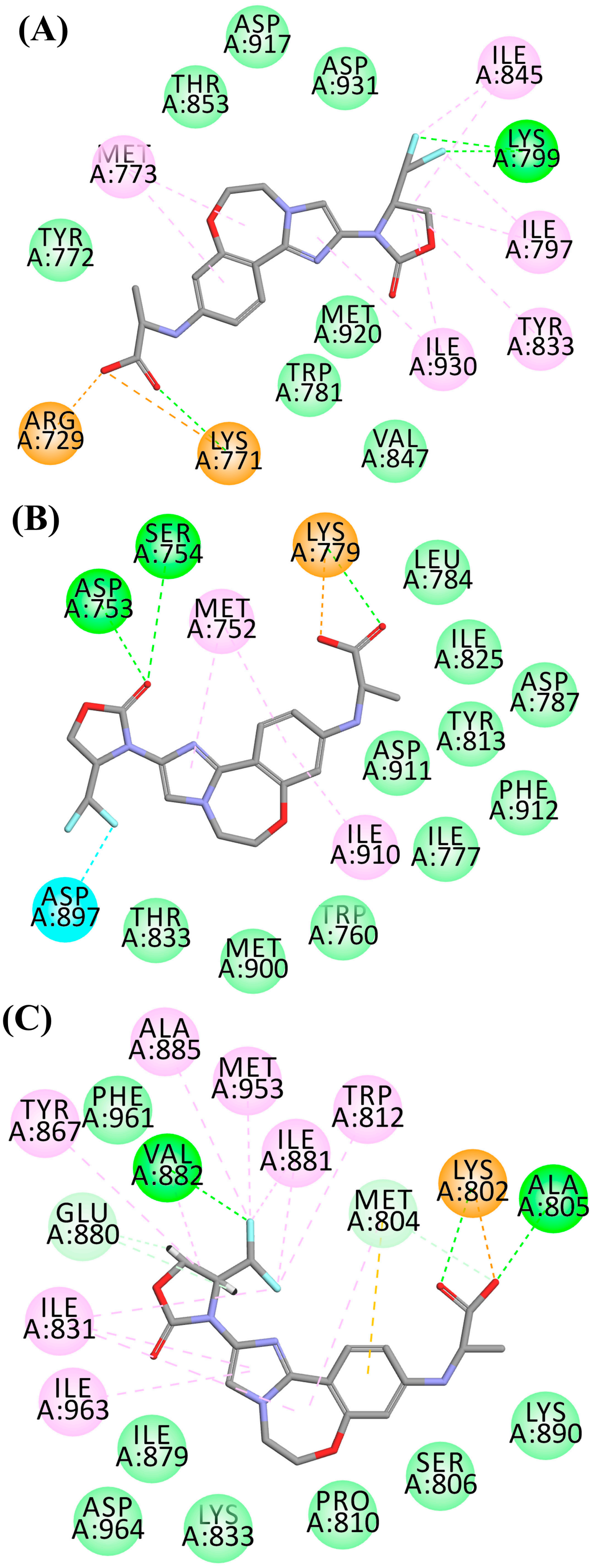

Figure S3. (A) The binding mode between M-288 and PI3Kβ. (B) The binding mode between M-288 and PI3Kδ. (C) The binding mode between M-288 and PI3Kγ.

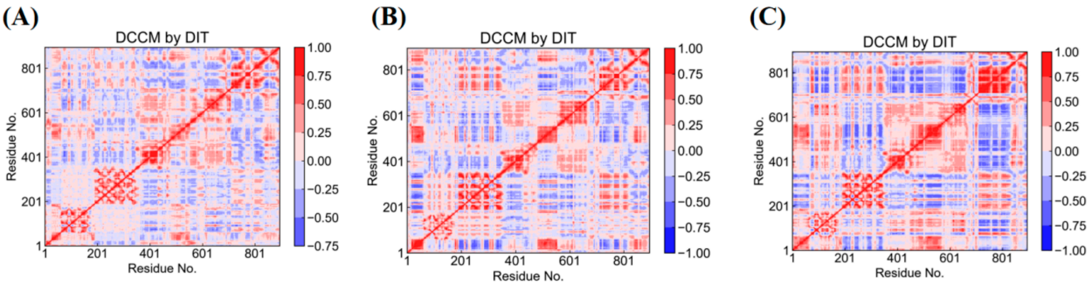

Figure S4. DCCM for the Cα fluctuations and their average positions of PI3Kα (a), PI3Kα-M-288 (b) and PI3Kα-inavolisib (c) systems. Red represents positive correlations, whereas blue represents negative correlations.

Table S1. The -CDOCKER energy of M-288/inavolisib with the ATP-binding pocket of PI3Kα/β/δ/γ.

Name	PI3Kα (kcal/mol)	PI3Kβ (kcal/mol)	PI3Kδ (kcal/mol)	PI3Kγ (kcal/mol)
M-288	45.6221	33.1794	27.0735	36.5349
Inavolisib	37.6246	29.6114	27.7060	27.3720

Table S2. The ADME of the top 13 compounds with docking scores.

**ID number**	**MW**[1]	**ALogP**[2]	**nRot**[3]	**nHA**[4]	**nHD**[5]
M-21	405.375	1.767	5	8	2
M-31	443.382	0.481	5	11	3
M-288	407.348	0.819	5	9	2
M-311	409.387	1.002	7	9	3
M-315	409.387	1.002	7	9	3
M-2170	403.359	2.116	4	8	2
M-329	421.358	1.565	6	11	4
M-338	406.383	2.6	6	8	1
M-352	423.324	2.433	7	10	0
M-360	422.343	1.202	5	11	4
M-2233	407.395	1.617	5	9	4
M-3105	406.383	2.401	7	8	1
M-41	422.406	2.151	5	8	3

1. MW: 0~500; **2.** ALogP: -2~6.5; **3.** nRot: 0~11; **4.** nHA: 0~10; **5.** nHD: 0~5.

Table S3. The binding free energy (kJ/mol) of PI3Kα with M-288/inavolisib, and its components between receptor and ligand.

Energy	PI3Kα-M-288	PI3Kα-Inavolisib
Van der Waals energy (kJ/mol)	-201.227	-205.627
Electrostatic energy (kJ/mol)	-118.633	-42.026
Polar solvation energy (kJ/mol)	232.627	169.412
Nonpolar solvation energy (kJ/mol)	-23.547	-23.517
Total binding energy (kJ/mol)	-110.780	-101.758
TΔS (kJ/mol)	17.307	14.038
Total binding free energy (kJ/mol)	-93.473	-87.720

Table S4. Predicted metabolites and metabolic pathways of M-288.

Name	priority_score	reaction_type	metabolite_smiles
1	0.984	O-glucuronidation_(aliphatic_carboxyl)	C[C@H](Nc1ccc2c(c1)OCCn1cc(N3C(=O)OC[C@H]3C(F)F)nc1-2)C(=O)OC1OC(C(=O)O)C(O)C(O)C1O
2	0.248	aliphatic_hydroxylation_(tert_carbon_next_to_SP2)	C[C@](O)(Nc1ccc2c(c1)OCCn1cc(N3C(=O)OC[C@H]3C(F)F)nc1-2)C(=O)O
3	0.248	decarboxylation	CCNc1ccc2c(c1)OCCn1cc(N3C(=O)OC[C@H]3C(F)F)nc1-2
4	0.162	aliphatic_hydroxylation_(tert_carbon_next_to_SP2)	C[C@H](Nc1ccc2c(c1)OCCn1cc(N3C(=O)OC[C@@]3(O)C(F)F)nc1-2)C(=O)O
5	0.162	hydrolysis_(urea_or_carbonate)	C[C@H](Nc1ccc2c(c1)OCCn1cc(N(C(=O)O)[C@@H](CO)C(F)F)nc1-2)C(=O)O
6	0.162	aliphatic hydroxylation	C[C@H](Nc1ccc2c(c1)OCCn1cc(N3C(=O)OC(O)[C@H]3C(F)F)nc1-2)C(=O)O
7	0.162	oxidative ether cleavage to one alcohol and one aldehyde/ketone	C[C@H](Nc1ccc2c(c1)OCCn1cc(N(C(=O)O)[C@@H](C=O)C(F)F)nc1-2)C(=O)O
8	0.159	N-dealkylation	Nc1ccc2c(c1)OCCn1cc(N3C(=O)OC[C@H]3C(F)F)nc1-2
9	0.159	N-dealkylation	CC(=O)C(=O)O
10	0.159	amine hydroxylation	C[C@@H](C(=O)O)N(O)c1ccc2c(c1)OCCn1cc(N3C(=O)OC[C@H]3C(F)F)nc1-2
11	0.148	hydrolysis_(urea_or_car	C[C@H](Nc1ccc2c(c1)OCCn1cc

		bonate)	(N[C@@H](COC(=O)O)C(F)F)nc1-2)C(=O)O
12	0.148	N-oxidation	C[C@H](Nc1ccc2c(c1)OCCn1cc([N+]3([O-])C(=O)OC[C@H]3C(F)F)nc1-2)C(=O)O
13	0.132	aromatic_hydroxylation_(ortho_to_2_substituents)	C[C@H](Nc1ccc2c(c1O)OCCn1cc(N3C(=O)OC[C@H]3C(F)F)nc1-2)C(=O)O
14	0.132	aliphatic hydroxylation	C[C@H](Nc1ccc2c(c1)OCCn1cc(N3C(=O)OC[C@H]3C(O)(F)F)nc1-2)C(=O)O
15	0.132	oxidative dehalogenation alkyl	C[C@H](Nc1ccc2c(c1)OCCn1cc(N3C(=O)OC[C@H]3C(=O)F)nc1-2)C(=O)O
16	0.116	O-dealkylation_(aromatic)	C[C@H](Nc1ccc(-c2nc(N3C(=O)OC[C@H]3C(F)F)cn2CCO)c(O)c1)C(=O)O
17	0.116	aliphatic_hydroxylation_(sec_carbon_next_to_SP2,in_a_ring)	C[C@H](Nc1ccc2c(c1)OCC(O)n1cc(N3C(=O)OC[C@H]3C(F)F)nc1-2)C(=O)O
18	0.116	N-dealkylation_(nCH2)	C[C@H](Nc1ccc(-c2nc(N3C(=O)OC[C@H]3C(F)F)c[nH]2)c(OCCO)c1)C(=O)O
19	0.116	oxidative ether cleavage to one alcohol and one aldehyde/ketone	C[C@H](Nc1ccc(-c2nc(N3C(=O)OC[C@H]3C(F)F)cn2CC=O)c(O)c1)C(=O)O
20	0.116	aliphatic hydroxylation	C[C@H](Nc1ccc2c(c1)OC(O)Cn1cc(N3C(=O)OC[C@H]3C(F)F)nc1-2)C(=O)O
21	0.116	N-oxidation	C[C@H](Nc1ccc2c(c1)OCC[N+]1([O-])C=C(N3C(=O)OC[C@H]3C(F)F)N=C21)C(=O)O
22	0.116	N-glucuronidation_(aniline_NH1-R)	C[C@@H](C(=O)O)N(c1ccc2c(c1)OCCn1cc(N3C(=O)OC[C@H]3C(F)F)nc1-2)C1OC(C(=O)O)C(O)C(O)C1O
23	0.116	N-dealkylation	C[C@H](Nc1ccc(-c2nc(N3C(=O)OC[C@H]3C(F)F)c[nH]2)c(OCC=O)c1)C(=O)O
24	0.104	aliphatic hydroxylation	O=C(O)[C@H](CO)Nc1ccc2c(c1)OCCn1cc(N3C(=O)OC[C@H]3C(F)F)nc1-2
25	0.08	N-dealkylation	C[C@H](Nc1ccc2c(c1)OCCn1cc(NC(=O)OCC(=O)C(F)F)nc1-2)

			C(=O)O
26	0.068	N-oxidation_(-N=)	C[C@H](Nc1ccc2c(c1)OCCn1cc(N3C(=O)OC[C@H]3C(F)F)[n+]([O-])c1-2)C(=O)O
27	0.06	aromatic hydroxylation	C[C@H](Nc1ccc2c(c1)OCCn1c-2nc(N2C(=O)OC[C@H]2C(F)F)c1O)C(=O)O
28	0.05	deformylation	O=CO
29	0.05	deformylation	C=CNc1ccc2c(c1)OCCn1cc(N3C(=O)OC[C@H]3C(F)F)nc1-2
30	0.044	N-glucuronidation_(aromatic_=n-)	C[C@H](Nc1ccc2c(c1)OCCn1cc(N3C(=O)OC[C@H]3C(F)F)[n+](C3OC(C(=O)O)C(O)C(O)C3O)c1-2)C(=O)O
31	0.032	carbamate cleavage	O=C=O
32	0.032	alkyl dehydrogenation	C[C@H](Nc1ccc2c(c1)OCCn1cc(-n3c(C(F)F)coc3=O)nc1-2)C(=O)O
33	0.032	carbamate cleavage	C[C@H](Nc1ccc2c(c1)OCCn1cc(N[C@@H](CO)C(F)F)nc1-2)C(=O)O
34	0.032	aromatic_hydroxylation_(para_to_oxygen)	C[C@H](Nc1cc2c(cc1O)-c1nc(N3C(=O)OC[C@H]3C(F)F)cn1CCO2)C(=O)O
35	0.032	dehydration of N-C bond	CC(=Nc1ccc2c(c1)OCCn1cc(N3C(=O)OC[C@H]3C(F)F)nc1-2)C(=O)O
36	0.032	alkyl dehydrogenation	C=C(Nc1ccc2c(c1)OCCn1cc(N3C(=O)OC[C@H]3C(F)F)nc1-2)C(=O)O
37	0.032	dehydration of N-C bond	CC(=[NH+]c1ccc2c(c1)OCCn1cc(N3C(=O)OC[C@H]3C(F)F)nc1-2)C(=O)O
38	0.026	alkyl dehydrogenation	C[C@H](Nc1ccc2c(c1)OCCn1cc(N3C(=O)OCC3=C(F)F)nc1-2)C(=O)O
39	0.023	oxidation of 4-substituted anisole to quinone	CCn1cc(N2C(=O)OC[C@H]2C(F)F)nc1C1=CC(=O)C(N[C@@H](C)C(=O)O)=CC1=O
40	0.023	alkyl dehydrogenation	C[C@H](Nc1ccc2c(c1)OC=Cn1cc(N3C(=O)OC[C@H]3C(F)F)nc1-2)C(=O)O
41	0.016	dehydration of N-C bond	C[C@H](Nc1ccc2c(c1)OCCn1cc([N+]3=C(C(F)F)COC3=O)nc1-2

			)C(=O)O
42	0.012	aromatic hydroxylation	C[C@H](Nc1cc(O)c2c(c1)OCCn1cc(N3C(=O)OC[C@H]3C(F)F)nc1-2)C(=O)O
